# HyEpiSeiD: a hybrid convolutional neural network and gated recurrent unit model for epileptic seizure detection from electroencephalogram signals

**DOI:** 10.1186/s40708-024-00234-x

**Published:** 2024-08-21

**Authors:** Rajdeep Bhadra, Pawan Kumar Singh, Mufti Mahmud

**Affiliations:** 1 Department of Information Technology, Jadavpur University, Jadavpur University Second Campus, Plot No. 8, Salt Lake Bypass, LB Block, Sector III, Salt Lake City, 700 106 Kolkata, West Bengal India; 2 Metharath University, 99, Moo 10, Bang Toei, Sam Khok, 12160 Pathum Thani, Thailand; 3 Department of Computer Science, Nottingham Trent University, Nottingham, NG11 8NS UK; 4 Medical Technologies Innovation Facility, Nottingham Trent University, Nottingham, NG11 8NS UK; 5 Computing and Informatics Research Centre, Nottingham Trent University, Nottingham, NG11 8NS UK

**Keywords:** Epileptic seizure detection, Electroencephalogram signals, HyEpiSeiD, Convolutional neural network, Gated recurrent unit, Epilepsy UCI dataset, Mendeley dataset

## Abstract

Epileptic seizure (ES) detection is an active research area, that aims at patient-specific ES detection with high accuracy from electroencephalogram (EEG) signals. The early detection of seizure is crucial for timely medical intervention and prevention of further injuries of the patients. This work proposes a robust deep learning framework called HyEpiSeiD that extracts self-trained features from the pre-processed EEG signals using a hybrid combination of convolutional neural network followed by two gated recurrent unit layers and performs prediction based on those extracted features. The proposed HyEpiSeiD framework is evaluated on two public datasets, the UCI Epilepsy and Mendeley datasets. The proposed HyEpiSeiD model achieved 99.01% and 97.50% classification accuracy, respectively, outperforming most of the state-of-the-art methods in epilepsy detection domain.

## Introduction

Epilepsy is an abnormal brain condition caused by various factors, which has affected many people all over the world. It is a brain disease that causes frequent and unpredictable disruptions to normal brain activity. It leads to various symptoms like uncontrollable jerking, unconsciousness, etc. On the other hand, epilepsy seizure refers to a sudden, uncontrolled, and abnormal electrical disturbance in the brain that leads to a wide range of symptoms or behaviors. According to the World Health Organization (WHO), yearly, over 50 million people over different age groups and residential backgrounds are affected by epilepsy [1–3] disease, out of whom many people die for lack of proper treatment. A report shows that there is a significant shortage of neurologists [4] who can treat this kind of disease [5–7]. Therefore, automating the process of epilepsy seizure detection will help us to aid neurologists and allied health providers in the treatment of such diseases.

Electroencephalographic (EEG) [8] is a method that aims to measure the electrical activity of different regions in the human brain [9]. It was first introduced by Hans Berger, who aimed to study the human brain. EEG is beneficial in the diagnosis of different kinds of brain disorders. During epilepsy seizures, EEG data of patients’ brains behaves differently than normal brain conditions. A detailed study of the EEG signals of different patients during epilepsy seizures helps us identify the specific characteristics of those signals that occur only during epilepsy seizures. In this literature, we have proposed a deep learning framework that can learn features from different EEG data of epilepsy patients by itself and accurately predict the epileptic activity of the brain from unknown EEG data. There are many other physiological methods [10–12] to get information about epilepsy other than EEG. However, EEG provides a noninvasive biophysical examination method for medical experts to study the characteristics of epilepsy. It gives us much more detailed information on the epilepsy condition of the patients. Most of the other physiological methods cannot offer as much detailed information about epilepsy as EEG can. Therefore, our study chose EEG signal data of patient’s brain activity for our epilepsy detection task.

Lately, Artificial Intelligence (AI), notably machine learning (ML) and deep learning (DL), have drawn considerable attention from researchers, spurring contributions across various fields and demanding research tasks including anomaly detection [13–15], signal analysis [16–28], neurodevelopmental disorder assessment and classification focusing on autism [29–37], neurological disorder detection and management [38–44], supporting the detection and management of the COVID-19 pandemic [45–52], elderly monitoring and care [53], cyber security and trust management [54–59], ultrasound image [60], various disease detection and management [61–68], smart healthcare service delivery [69–71], text and social media mining [72–74], understanding student engagement [75, 76], etc. Epilepsy detection problem has been tackled mainly by traditional machine learning approaches [77–79], which consists of extracting quality features followed by classifying the feature set whether it is epileptic. Unlike deep learning, it requires manual intervention of feature engineering, which is subject to several rounds of trial and error for optimal performance. The performance of these methods varies with different datasets.

Therefore, we have proposed a deep learning framework [80, 81] for robust classification of EEG datasets. We have chosen the deep learning paradigm since it eliminates the trouble of hand-crafted feature extraction and automatically generates all informative self-learned feature sets [25, 82].

We have chosen one-dimensional convolutional neural networks (CNN) and a special kind of recurrent neural network (RNN) for our study. Our model framework consists of 4 1D convolutional layers, a fully connected layer, and 2 Gated Recurrent Units (GRU) [83]. The last layer is a Softmax layer, which does the final prediction. Our model pipeline is further evaluated on two different public datasets, i.e. (i) UCI Epilepsy Dataset [84], (ii) Mendeley Dataset by Renuka Khati [85]. which outperforms most of the state-of-the-arts. Initially, we ran our model excluding the GRU component. Including GRU in our model pipeline has improved the performance metrics results by a significant margin. Results of both model pipelines (including and excluding GRU) have been compared in the Results section.

## Related work

Several studies focused on epileptic seizure recognition from EEG signals, employing different techniques and approaches for accurately identifying and classifying seizures into their respective classes. This section has reviewed some recent state-of-the-art methods in this field. The problem of EEG-based epileptic seizure recognition has been broadly investigated over the past three decades. Initially, only traditional machine learning algorithms have been used in epilepsy detection [77–79]. Later, deep learning methods have also come into the picture. Most deep learning state-of-the-art methods [86–88] give more robust classification results than traditional ones. Some such state-of-the-art methods are reviewed and summarised here.

Chandaka et al. [89] proposed a method where three statistical features were computed using EEG cross-correlation coefficients and presented them in a feature vector in a support vector machine(SVM). This method has achieved a decent accuracy of 95.96% in detecting epilepsy seizures from EEG data. Furthermore, Aarabi et al. [90] proposed a deep learning method in which features are extracted from the time, frequency, wavelet domain and fed into a back-propagation neural network. This method has achieved a classification accuracy of 93.00%. In their proposed method, Yuan et al. [91] used Hursh exponent and entropy as a set of non-linear features and extreme learning machine (ELM) classifier. This method has led to good accuracy in epilepsy detection. Many authors have used wavelet transformation to extract representative features from EEG data. Subasi et al. [92] proposed another method where spectral components are used as an input in a mixture of experts. Spectral components are derived from wavelet transformation. This proposed method achieved a classification accuracy of 94.50%. Khan et al. [93] also have used wavelet transformation where wavelet coefficients at lower frequency range are processed to compute the representative features from EEG signals. This method gives around 91.80% classification accuracy. Kumar et al. [94] also proposed another wavelet transformation method where EEG signals are divided into five different frequency bands, then extracting a set of non-linear features and further feeding into a support vector machine (SVM) classifier. This method has achieved a moderate classification accuracy of 97.50%. Nicolaou et al. [95] used permutation entropy as a feature in epilepsy detection and employed a support vector machine (SVM). This method gives an average of 93.28% classification accuracy. In addition, Song et al. [96] also employed weighed permutation entropy features along with SVM to obtain a better classification result. This method also achieves around 97.25% classification accuracy. Some of the most recent works on this domain used combination of different models or statistical feature selection methods to ensure robust classification results.Rohan et al. [97] uses combination of an artificial neural network along with a XGBoost model for classification. It gives robust classification accuracy around 98.26%.Later, Shankar et al. [98] proposed a new method where artificial neural network model had been combined with principal component analysis statistical method.This state-of-the-art also achieved pretty well classification results on EEG data, around 97.55% accuracy.Similarly, Rahman et al. [99] also proposed a combination of Support Vector Machine and Multi-layer Perceptron classifier.This method achieves around 97.27% accuracy, 96.93% precision, 94.53% recall.Prakash et al. [100] tried something new and introduced Gated Recurrent Unit in his state-of-the-art.This method achieves remarkable around 98.84% classification accuracy.Later, Raibag et al. [101] used Support Vector Machine with Radial Basis Function kernel for epilepsy detection.In this method, firstly Principal Component Analysis has been performed for dimensionality reduction of EEG data, followed by employing a Support Vector Machine with Radial Basis Function kernel.This method gives around 96% classification accuracy.Osman et al. [102] proposed Self-organizing map along with Radial Basis Function kernel neural network for epilepsy detection.In this work, Self-organizing map(SOM) has been used for feature dimensionality reduction of raw EEG data.SOM converts high dimensional EEG data into a two dimentional map.Further, a Radial Basis Function neural network has been employed for further classification.This method gives around 97.47% classification accuracy. Upadhyaya et al. [103] introduced BAT algorithm on optimal feature selection from EEG data for epilepsy detection.This proposed method performs well and gives around 96.78% classification accuracy. Woodbright et al. [104] used convolutional neural networks and pooling layer to capture spatial and temporal features characteristics from EEG data.This method gives around 98.65% classification accuracy. Wang et al. [105] introduced rule-based classifier for epilepsy detection.In their work, firstly, noise reduction and signal normalization have been performed for preprocessing of EEG data, followed by employing a rule based classifier like Random Forest.Guha et al. [106] used artificial neural network for epilepsy detection.

We can see that most of the state-of-the-art methods currently present in the epilepsy recognition domain use either traditional machine learning algorithms or simple deep learning methods.There are very few recent works where hybrid combination of models or statistical feature selection methods have been used. Most state-of-the-art methods’ main focus relies on robust preprocessing of raw EEG data and not on proper model selection. Here, in this study, we are more focused on including a correct combination of models in our model pipeline so that it can give optimal results on any dataset. Since EEG signal data is a special type of time series data, we have decided to include a 1D convolutional neural network in our model pipeline. We know that RNN models generally perform well on sequential data. Since EEG signal data is a special sequential data type, we have also included a RNN in our model architecture. After several experiments, we have developed a hybrid CNN+GRU model that can give robust classification results on any EEG dataset.We have benchmarked the performance of our model framework with some of the most recent state-of-the-arts.

### Motivation behind using this framework

In our model framework, we have used a hybrid 1D-CNN and GRU combination for epilepsy detection from EEG data. Although, there are many other approaches that we had considered to tackle this problem, but at the end, we had decided to move with this framework.Most of the works that had been carried out in this field of epilepsy detection, mostly revolves around traditional machine learning algorithms, wavelet transformations,statistical feature analysis etc.Here, we have come around something out of the box methodologies which gives much more robust classification results. Moreover, there are many state-of-the arts which were evaluated only on one dataset.Although, those state-of-the-arts perform very well on that particular dataset, there is still a question on generalized behavior of those state-of-the-arts. Therefore, we have decided to evaluate our model framework on more than one dataset to ensure the acceptability of our model framework.

We know that EEG data is a special kind of time-series data.Since, we are working on time-series data, we have come up with an idea to use such models which gives better classification results with time-series data. We have experimented with several such models and finally, have decided to move with 1D CNN model as a part of our overall model framework.

We have experimented with many models and later decided to use a hybrid combination of those models which had been performing very well on the datasets. Finally, we have come up with our model framework, named as HyEpiSeiD, a hybrid combination of CNN and GRU. More detail implementation of our model pipeline is described in Sect. 3.

## Proposed HyEpiSeiD framework

**Fig. 1:**
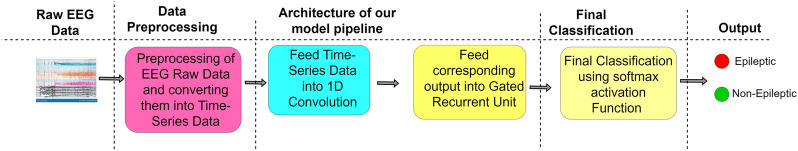
Pictorial Representation of different phases of our model pipeline for epilepsy seizure detection from raw EEG data

This section provides a detailed overview of our model pipeline for epilepsy detection from EEG signal data. Figure 1 gives an abstract overview of the different stages of our model pipeline and how each stage interacts with each other.

Our HyEpiSeiD model pipeline architecture consists of the following stages.

- Feeding the time series data in four consecutive 1D CNN.
- Self-learned features in 1D convolutional layers are then passed through a pooling layer.
- Further, a dropout layer is added after the pooling layer to prevent redundant features from propagating further.
- Now, to obtain better accuracy in our datasets, we have introduced GRU layers, a special type of RNN. It further improves our model’s performance in our model pipeline.
- Finally, a Softmax function is applied at the last layer in our model pipeline for final classification.

In our study, we have performed two types of classification: (i) 2-class classification, where we aim to check whether a seizure is epileptic, and (ii) 5-class classification, where the non-epileptic EEG signals are also further divided into different classes, leading the number to the classes becoming more than 2. We aim to classify all EEG signals into their respective classes.

Here, the datasets used in our study are already pre-processed and converted to time-series data. We are directly feeding them in our model architecture.

The stages of our model pipeline mentioned above are explained in detail in the following subsections.

### Feed time series data into 1D convolution

In our study, the datasets we have used are already pre-processed and converted into time-series data. We have used those time-series data directly as input into our model pipeline. The first stage of our model architecture consists of four 1D CNNs. These 1D CNNs extract self-learning features from the input vector. These self-learning features are further used for accurate classification. The architectures of four 1D CNNs used in our study are explained below in subsections.

**Fig. 2:**
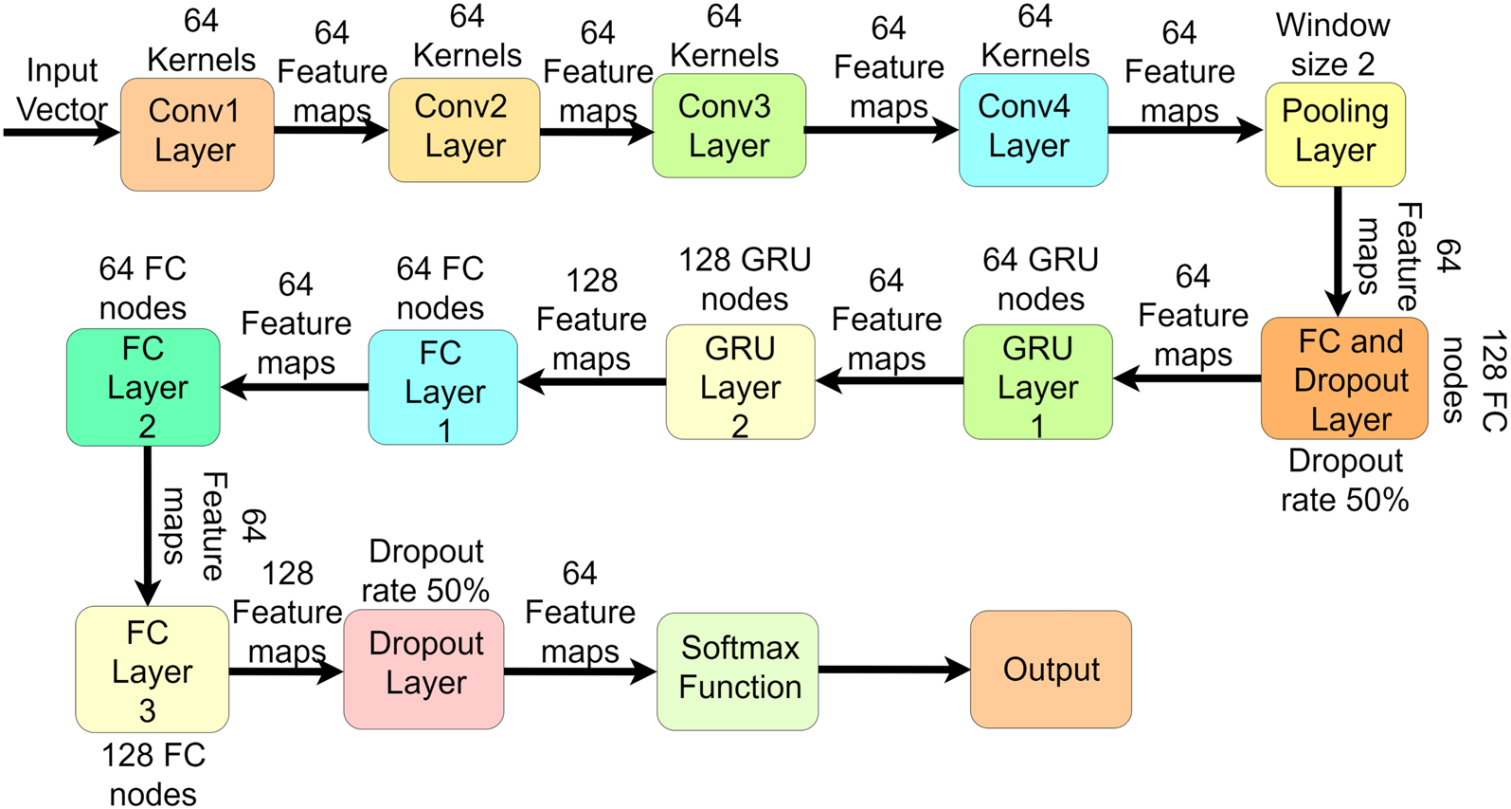
Detailed Architecture of our proposed HyEpiSeiD framework for Epilepsy seizure detection from EEG signals

We have used four 1D CNNs in our model pipeline, all with different architectures, shown in Fig. 2. The input vector of the time-series dataset is directly fed into the first 1D CNN of our model architecture. The first 1D CNN consists of 64 kernels, each with a size of 3$\times$1. This convolutional layer is followed by a Rectified Linear Unit (ReLU) activation function, which introduces non-linearity to our model pipeline. The mathematical definition of our 1D convolutional network is explained below.

1 $$\begin{aligned} y_j^l&= \textrm{ReLU}\left( \sum _{i=1}^{N_{l-1}}\left( \textrm{conv1D}\left( w_{i,j}^l ,\ x_i^{l-1}\right) + b_j^l \right) \right) \end{aligned}$$

where, $x_i^{l-1}$ represents the $i^{\text {th}}$ feature mapping inside the model’s (l-1) convolutional layer. $y_j^{l}$ represents the $j^{\text {th}}$ feature mapping inside the model’s $l^{\text {th}}$ convolutional layer, $N_{l-1}$ represents the total number of feature maps in $(l-1)^{\text {th}}$ layer, $w_{i,j}^l$ denotes the weight values of convolutional kernels used in the model, $b_j^{l}$ denotes the bias of $j^{\text {th}}$ feature mapping inside model’s $l^{\text {th}}$ layer. In the equation (1), *conv*1*D*() represents one-dimensional convolutional operation on trainable parameters. Finally, *ReLU* represents the ReLU operation, which introduces non-linearity in our model. The mathematical definition of the ReLU activation function is defined as follows.

2 $$\begin{aligned} \textbf{ReLU}({\textbf{x}}) = {\left\{ \begin{array}{ll} 0 &{} \text {if } x \le 0, \\ x &{} \text {if } x > 0. \end{array}\right. } \end{aligned}$$

The ReLU function only activates when the input signal value is greater than 0; otherwise, it returns 0. Thus, it introduces non-linearity in our model and allows us to understand the dataset’s non-linear patterns. After the convolution operation, it outputs 64 feature maps, which are again passed to the next CNN layer.

The second 1D convolutional layer takes these 64 feature maps as input and performs the feature learning process again as the previous convolution layer. The second 1D convolution layer consists of 64 kernels, each with a size of 3$\times$1. As before, the CNN layer is followed by a ReLU activation function. This layer outputs 64 feature maps, further fed into the next convolutional layer.

The third convolutional layer takes 64 feature maps as input from the previous layer and performs the same operation as the previous one. This 1D CNN layer consists of 64 kernels, each kernel of size 3$\times$1. This layer extracts useful features from input and outputs 64 feature maps. As with other previous CNN layers, this CNN layer is also followed by a ReLU activation function. This ReLU portion helps to identify non-linear patterns of data, which leads to more accurate classification. The outputted feature maps are again fed into the next CNN layer.

The fourth CNN layer consists of 64 kernels, each with a size of 3$\times$1. This is the last convolution layer used in our model framework. It takes 64 feature maps as input and outputs 64 feature maps. This layer is also followed by a ReLU activation function.

These are the model architectures of all 1D convolutional layers used in our framework. Using four 1D CNNs leads our model to extract the most insightful features, training them properly and propagating them throughout the rest of the layers of our model. Since we have converted our dataset into time-series data, we have decided to use 1D CNN since it generally gives good accuracy performance measures on time-series data. Finally, 64 feature maps, which the last CNN layers have outputted, are passed to the pooling layer for further operation.

### Feed CNN layer output into pooling layer

Finally, after four 1D CNN layers, the features are fed into the pooling layer. In our model framework, we have only used one pooling layer, which uses the Max pooling method. The 64 features from the last one-dimensional convolutional layer are directly passed into this pooling layer. The mathematical explanation of max pooling can be defined as below.

3 $$\begin{aligned} {\textbf{p}}_{i}^{a} = \max \left( {\textbf{p}}_{i}^{a_{1}}: a_{1} \le a_{1} < a_{1} + s \right) \end{aligned}$$

where, $p_i^a$ denotes the value carried by *a th *neuron inside *i th *feature obtained from the previous layer after the max pooling operation. $p_i^{a_1}$ denotes the value carried by $a_1$ th neuron inside *i th *feature. *max() *denotes the maximum neuron value within the pooling window. Here, s is the size of the pooling window. In our model, the size of the pooling window is set to 2, and the stride of the pooling window is also set to 2. This layer reduces the number of training parameters of the features, accelerates the training process and reduces the probability of overfitting.

After the Maxpooling operation, 64 feature mappings are outputted with reduced dimensions and further propagated throughout the model pipeline.

### Feed output features into a fully connected layer and dropout layer

After Maxpooling, we obtain 64 highly insightful feature maps as output. These output feature maps are passed through a fully connected layer (FC) followed by a dropout layer. The FC layer has 128 nodes, each with a ReLU activation function. All the nodes forward the input to the next layer. This FC layer is followed by a dropout layer where the dropout rate is 50%. This means that half of the features will not be propagated further and will be discarded. This layer maintains the number of quality features in the model and the discard of redundant features. It reduces the chances of overfitting problems in the model. After the dropout layer, half of the total features are further propagated to the next layer.

### Feed output features into gated recurrent units

**Fig. 3:**
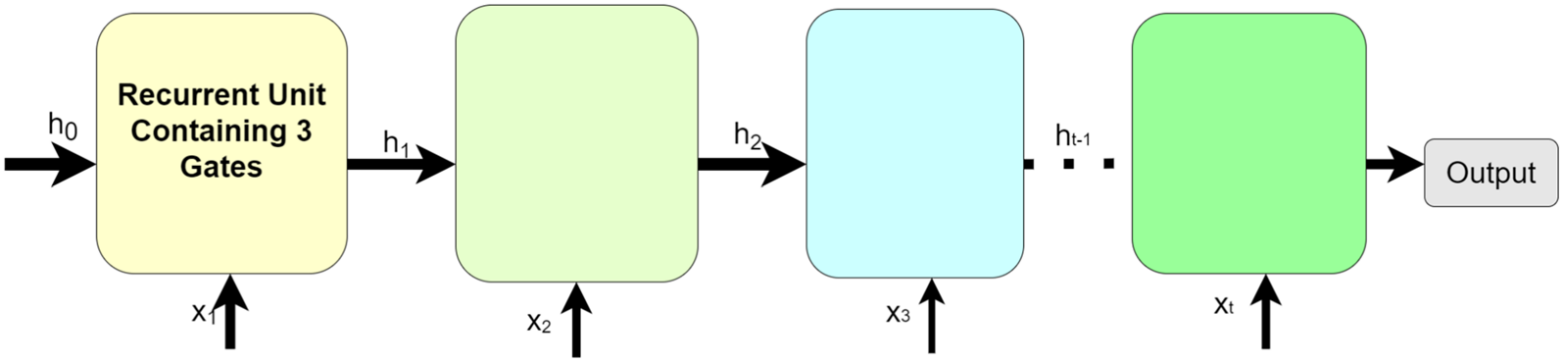
General architecture of an GRU recurrent neural network.The boxes denote nodes, the upward arrow indicate the previous input and the forward arrow indicates passing the output to the next node

After the dropout layer, half of the total features are fed into two GRUs. We have integrated RNN [107] with CNN in our model framework. Since RNN and CNN both give better performance results with time-series data, we have integrated them to optimise their performance. Here, we have used a special type of RNN, i.e. GRU. Two layers of GRU have been used next to the dropout layer in our model framework. The architecture of a GRU network is presented in Fig. 3. The first GRU layer consists of 64 nodes and returns a full sequence of outputs for each time stamp for our time series dataset. The second GRU layer has 128 nodes and also returns a full sequence of outputs for each time stamp. GRU is a special type of RNN that aims to perform better on sequential data, text and time-series data classification. We know that RNN can store the previously generated output in its hidden state and again use those stored outputs as input, allowing the network to capture sequential dependencies. Here, we have used GRU, a special type of RNN which uses gating mechanisms to perform different state operations inside the network selectively at different timestamps. These gating mechanisms control the flow of information in and out of the network. The GRU consists of three gating mechanisms: *(i)* reset gate, *(ii)* update gate, and *(iii)* current memory gate.

The *reset gate* checks how much of the past data, stored in the hidden state, needs to be discarded in the next iteration. This gate discards all the unnecessary past information stored in the hidden state memory.

The *update gate* checks how much past data needs to be reused in the next iteration. It also updates the hidden state with new input data. This gate finally determines the output of GRU.

*Current memory gate* is a subpart of the reset gate. This gate transforms the original input into zero mean non-linear input.

The mathematical representation for each gate of GRU is given below.

#### Reset gate

4 $$\begin{aligned} r_t = \sigma \left( w_r *\left( h_{t-1},\ x_t\right) \right) \end{aligned}$$

where, $r_t$ denotes the state after the reset gate operation, $w_r$ denotes the learnable weight matrix, $x_t$ represents the input at the timestamp t and $h_{t-1}$ represents the hidden state value at timestamp $(t-1)$.

#### Update gate

5 $$\begin{aligned} z_t = \sigma \left( w_z *\left( h_{t-1},\ x_t\right) \right) \end{aligned}$$

where, $z_t$ denotes the state after the update gate operation, $w_z$ denotes the learnable weight matrix, $x_t$ represents the input value at the timestamp *t* and $h_{t-1}$ represents the hidden state value at timestamp $(t-1)$.

#### Candidate hidden state

6 $$\begin{aligned} h_{t_1} = \tanh \left( w_h *\left( r_t *h_{t-1},\ x_t\right) \right) \end{aligned}$$

where, $h_{t-1}$ represents the candidate’s hidden state. $w_h$ represents the learnable weight matrix, $x_t$ represents the input at timestamp t, $h_{t-1}$ represents the hidden state at timestamp $(t-1)$ and $r_t$ represents the state after reset gate operation.

#### Hidden state

7 $$\begin{aligned} h_t = (1 - z_t) *h_{t-1} + z_t *h_{t_1} \end{aligned}$$

where, $h_t$ represents the candidate hidden state, $h_{t-1}$ represents the hidden state at the timestamp $(t-1)$, and $z_t$ represents the state after the update gate operation. In all the above equations, *sig*() denotes the sigmoid activation function and *tanh*() denotes the hyperbolic tangent activation function.

### Final classification

The outputs obtained from two GRU layers are further passed into three fully connected layers and a dropout layer, followed by a softmax activation function that performs the final classification. The architectures of all FC layers used in the final layer of our model framework are described in the following subsections. The first FC layer consists of 64 nodes, each with a ReLU activation function. This layer forwards the features to the next FC layer. The second FC layer also consists of 64 nodes, each with ReLU as its activation function. This layer takes input from the previous FC layer and forwards it to the next FC layer. The third and final FC layer consists of 128 nodes with a ReLU activation function. From this layer, 128 feature maps are fed into a dropout layer where 50% of these features are discarded to avoid overfitting problems. Finally, the rest of the features are passed into *Softmax* activation function, which performs the final classification.

## Experimental design

Here, we explain the experimental design of our pipeline, the datasets that we used to evaluate our proposed model framework, and also the performance results we obtained on those datasets while evaluating our model. Finally, we list some comparisons of our model framework with existing state-of-the-art methods in epilepsy detection.

### Datasets

Our proposed HyEpiSeiD framework is evaluated on two publicly available datasets. The two datasets used in our model evaluation are listed below.

1. UCI Epilepsy dataset
2. Mendeley dataset by Renuka Khati

#### UCI Epilepsy dataset

This dataset consists of 5 folders, each containing 100 files. Each file consists of the recording of the brain activity of one person. The brain activity for each person has been recorded for 23.6 s. These raw data files have already been pre-processed for our study. Each person’s brain activity data has been sampled into 4097 data points. The data points are further divided for each individual second timestamp. Therefore, for each second timestamp, only 178 data points are present. Hence, in the already pre-processed.csv file, there are 178 columns, each representing a particular feature value of EEG data at a specific timestamp. This dataset has five classes in total. The meaning of each class label is mentioned below.

**Class 5**—Label 5 indicates that the person’s eyes are open, meaning the patient had their eyes open when the brain’s EEG signal was being recorded.

**Class 4**—Label 4 indicates that the person’s eyes are closed, which means the patient had their eyes closed when the EEG signal had been recorded.

**Class 3**—Label 3 indicates that the region of the tumour in the brain was identified after recording the EEG activity from the healthy brain area.

**Class 2**—Label 2 indicates that the EEG signal was recorded from where the tumour was located.

**Class 1**—This label indicates the recording of seizure activity.

Class-wise data distribution in this UCI Epilepsy dataset is tabulated in Table 1. Here, we have performed two kinds of classification:

**2-class classification**—In our UCI epilepsy dataset, only class 1 represents epilepsy seizure, and the rest do not. Therefore, in the 2-class classification, we have only classified whether a set of data points at a particular timestamp represents an epileptic seizure. Hence, classes 2,3,4,5 of the original dataset are merged here and transformed into one class. Class-wise distribution of data in this UCI Epilepsy dataset for 2 class classification is tabulated in Table 1.

**5-class classification**—Here, the number of classes for the classification task is 5. We are not only limited to performing the classification of EEG signals into epileptic and non-epileptic. Here, non-epileptic EEG signal data are further classified into four classes. Class-wise distribution of data in this UCI Epilepsy dataset for 5-class classification is tabulated in Table 2.

**Table 1:** Class-wise distribution of the datasets we have considered for evaluating our model’s performance for 2 class classifications

Dataset	Class	Label	No. of samples
UCI Epilepsy	0	Epileptic	2300
	1	Non-Epileptic	9200
Mendeley dataset by Renuka Khati	0	Epileptic	100
	1	Non-Epileptic	100

#### Mendeley dataset by renuka khati

This dataset consists of 3 folders named *(i) set b, (ii) set d, (iii) set e*. Each folder contains text (.txt) files containing EEG signal time series data for different persons. Set b folder contains all those normal EEG signal data. Set d folder contains all preictal EEG signal data. Set e contains all epileptic EEG signal time-series data. This dataset has been pre-processed and converted into a comma-separated value (CSV) file. Each CSV file row gives the data point at a particular timestamp. This pre-processed CSV contains 11 features. Unlike the UCI Epilepsy dataset, there are only two classes in the pre-processed version of the Mendeley dataset. Therefore, we have only performed 2-way classification for this dataset.

**Table 2:** Class-wise distribution of the UCI Epilepsy dataset we have considered for evaluating our model’s performance for 5-class classification

Dataset	Class	Label	No. of samples
	1	Epileptic	2300
	2	tumour	2300
UCI Epilepsy	3	tumour Healthy	2300
	4	Eyes Closed	2300
	5	Eyes Opened	2300

### Hyperparameters values and train-test split details of our model framework

Our proposed HyEpiSeiD model pipeline has been implemented using the TensorFlow toolbox [108] in Python. UCI Epilepsy dataset is split into an 80:20 train-test split ratio for training. Our model has been trained for 20 epochs in this dataset with a mini-batch descent optimiser and a learning rate of 0.001. The batch size is set here as 32.

Like the UCI Epilepsy dataset, Renuka Khati’s Mendeley dataset is also split into an 80:20 train-test ratio. Our model has been trained for 20 epochs in this dataset with a mini-batch descent optimiser and a learning rate of 0.001. The batch size is set here as 32.

### Performance evaluation metrics

Here, four metrics are used to determine the evaluation performance of our model framework on these datasets. These metrics are (i) Accuracy, (ii) Precision, (iii) Recall, and (iv) F1-score, respectively. Mathematical representations of these evaluation metrics are mentioned below.

8 $$\begin{aligned} \text {Precision} = \frac{C_{ii}}{\sum _{j=1}^{N} C_{ji}} \times 100 \end{aligned}$$

9 $$\begin{aligned} \text {Accuracy} = \frac{\sum _{i=1}^{N} C_{ii}}{\sum _{i=1}^{N} \sum _{j=1}^{N} C_{ij}} \times 100 \end{aligned}$$

10 $$\begin{aligned} \text {Recall} = \frac{C_{ii}}{\sum _{j=1}^{N} C_{ij}} \times 100 \end{aligned}$$

11 $$\begin{aligned} \text {F1-score} = \frac{2 \cdot \text {Precision} \cdot \text {Recall}}{\text {Precision} + \text {Recall}} \times 100 \end{aligned}$$

where, *N* denotes the total number of classes in the dataset, and $C_{ij}$ denotes the matching value of $i^{\text {th}}$ datapoint, which belongs to the $j^{\text {th}}$ class. We have evaluated all these metric values of our proposed model on these datasets.

**Table 3:** Classification results (considering 2-class and 5-class labels) of our proposed HyEpiSeiD framework in UCI Epilepsy dataset

Dataset	Metric	Performance	
		2-Class	5-Class
UCI	Accuracy (%)	99.01	78.74
	Precision (%)	99.01	79.38
	Recall (%)	99.04	78.73
	F1-Score (%)	99.02	78.52

## Results and discussion

We have evaluated our proposed HyEpiSeiD framework on two publicly available datasets, namely UCI Epilepsy and the Mendeley dataset by Renuka Khati. We have calculated all of the evaluation metrics, i.e., accuracy, precision, recall, and f1-score on these datasets. Later, we compared these results with some existing state-of-the-art results. The results we have obtained on these two datasets are briefly explained in the following two sections.

### Results on UCI epilepsy dataset

Our HyEpiSeiD model framework has been run on this dataset for 30 epochs with an 80:20 train-test split ratio. We have performed two types of classification as mentioned in the section 4.1.1 in this dataset. The evaluation results for both kinds of classification are tabulated in Table 3.

In our model framework, we have actually integrated GRU with a 1D convolutional neural network. To better understand the effect of using GRU and whether it is improving the model’s performance, we have also compared the results of our model with GRU and without GRU. It means that we once evaluated our model on this dataset using the GRU layers along with 1D CNN layers, and later, we removed the GRU layer from our model pipeline and again evaluated the model in the same dataset.

**Table 4:** 2-class and 5-class classification comparison of our HyEpiSeiD model pipeline with GRU and without GRU for the UCI Epilepsy dataset

Class	Model	Accuracy (%)	Precision (%)	Recall (%)	F1-Score (%)
2-class	With GRU	99.01	99.01	99.04	99.02
	Without GRU	98.00	97.91	98.00	97.98
5-class	With GRU	78.34	79.38	78.73	78.52
	Without GRU	73.13	73.26	73.13	72.79

**Fig. 4:**
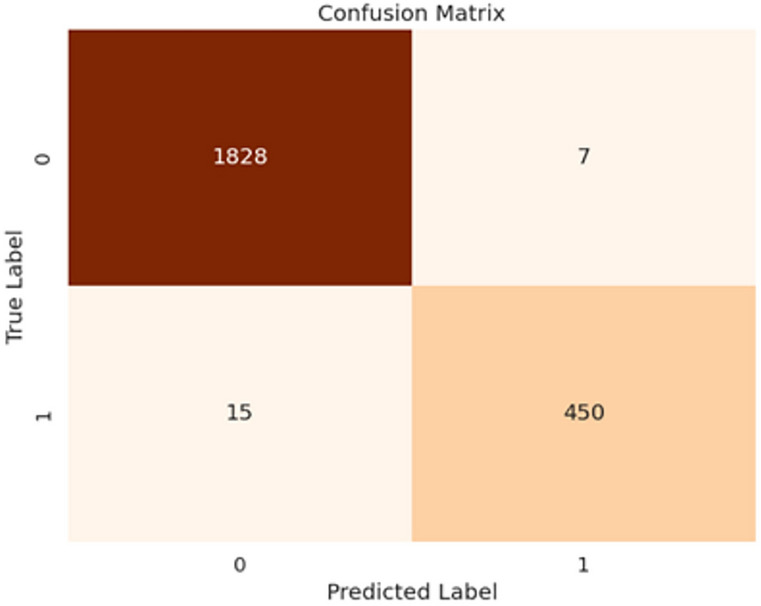
Confusion matrix of our proposed HyEpiSeiD model on UCI Epilepsy dataset for 2-class classification problem

**Fig. 5:**
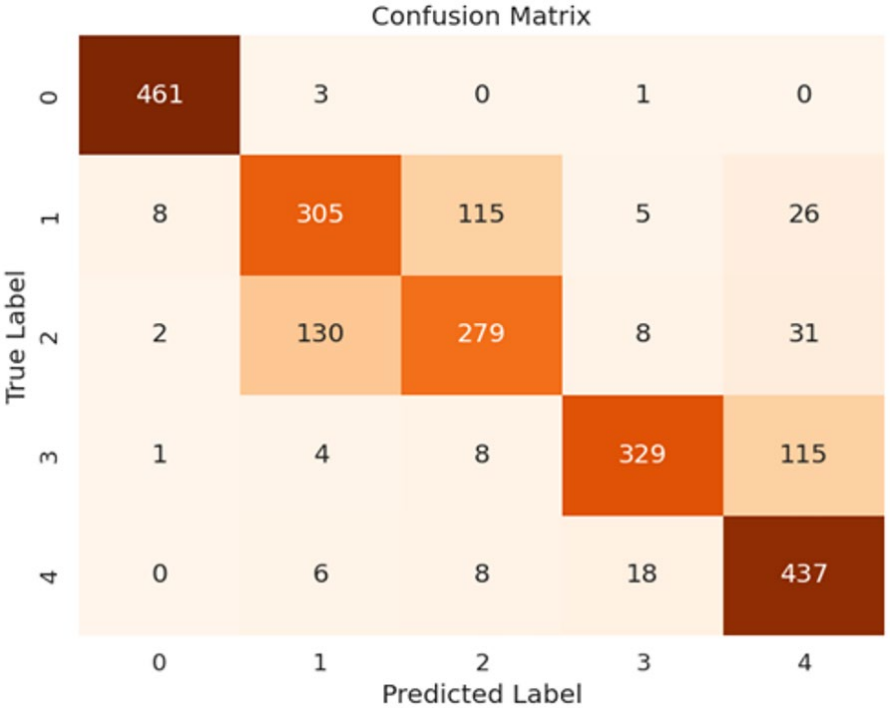
Confusion matrix of our proposed HyEpiSeiD model on UCI Epilepsy dataset for 5-class classification problem

**Fig. 6:**
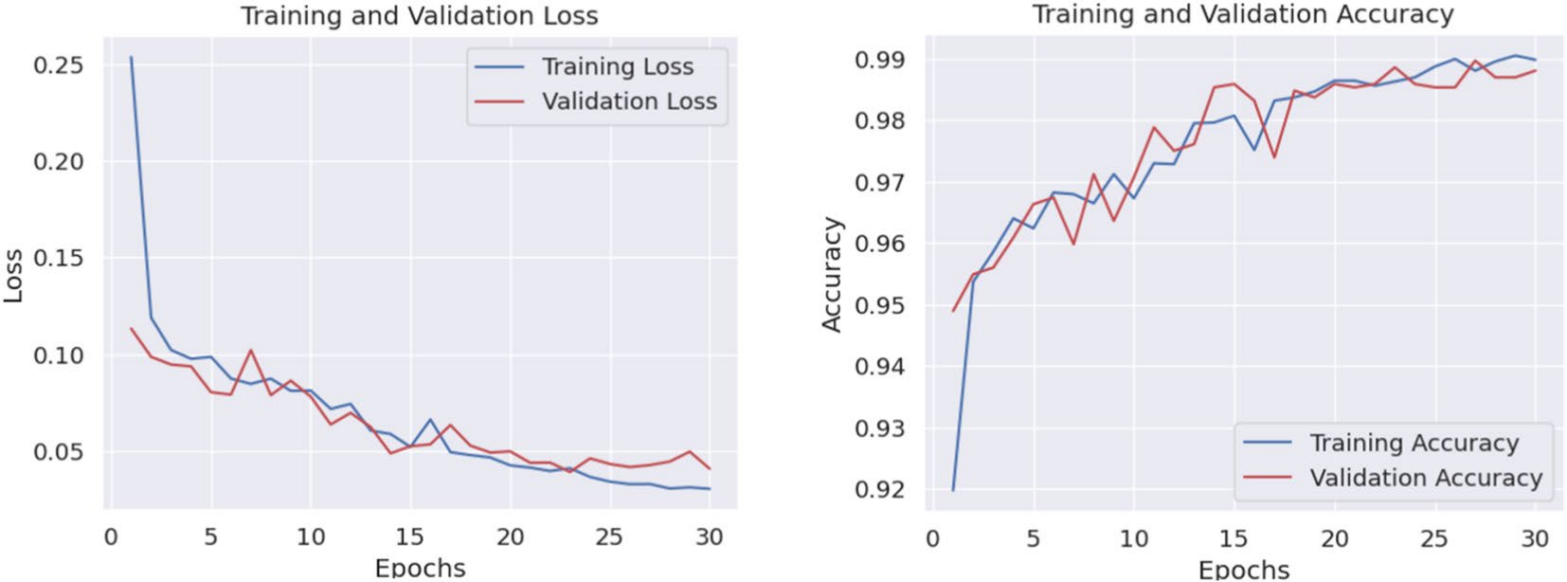
loss vs epoch and accuracy vs epoch curve of our proposed HyEpiSeiD model for UCI Epilepsy dataset in 2-class classification problem

The results obtained from both cases are compared in Table 4. While evaluating our model, we have split 20% of our total training data as validation data. We can see that, for 2-class classification, our model framework shows 99.05% accuracy on training data and 98.87% accuracy on validation data. Finally, our proposed framework shows 99.01% accuracy, 99.01% precision, 99.04% recall and 99.02% f1-score on test data in 2-class classification. The confusion matrix gives the overall performance of our proposed framework on the dataset by showing the model’s true positive, true negative, false positive and false negative values. For both classifications, either 2-class or 5-class, we can observe that the true positive and true negative values are very high, leading our model framework to give robust classification results regarding every possible evaluation metric, i.e. precision, recall, f1-score, and accuracy.

**Fig. 7:**
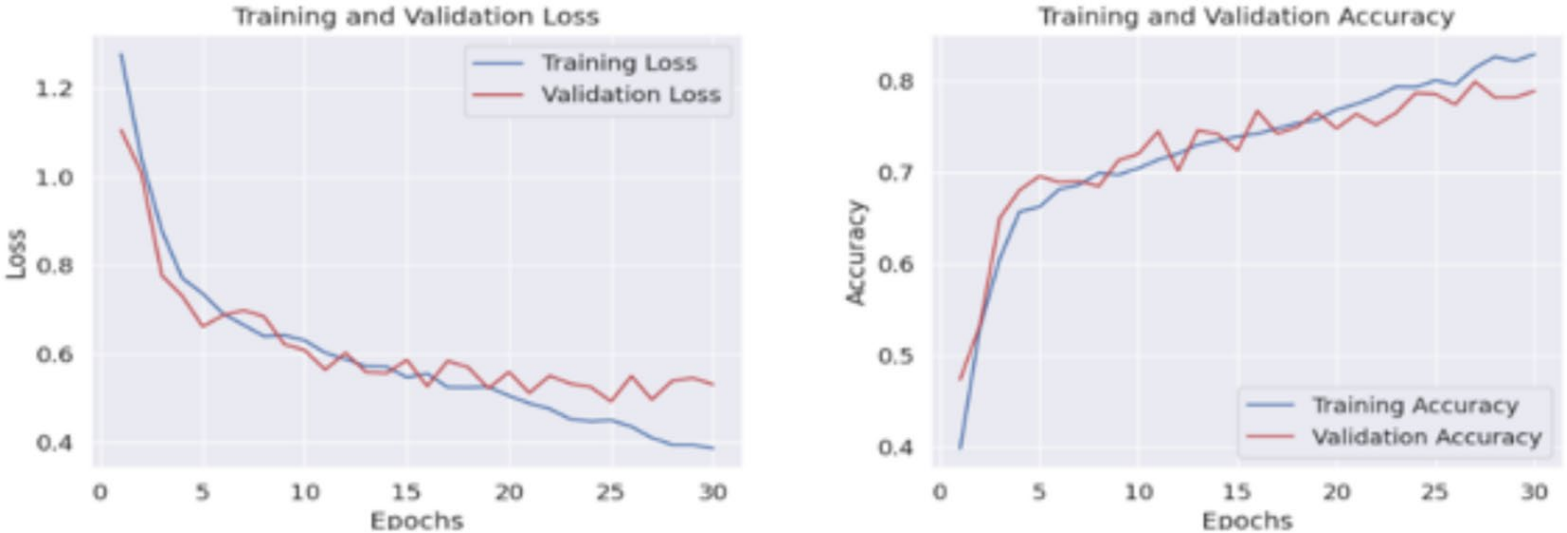
Loss vs epoch and accuracy vs epoch curve of our proposed HyEpiSeiD model on UCI Epilepsy dataset for 5-class classification problem

Figure 4 shows the confusion matrix obtained in 2-class classification, whereas Fig. 5 shows the confusion matrix obtained in 5-class classification. Similarly, for 5-class classification, we observe that training data shows 79.57% accuracy and the validation data shows 77.39% accuracy. In the end, our framework gives 78.74% accuracy, 79.34% precision, 78.73% recall, and 78.52% f1-score value on the test dataset. Figure 6 shows the loss versus epoch and accuracy versus epoch curves during training for 2-class classification on the UCI Epilepsy dataset. Figure 7 shows the loss vs epoch and accuracy vs epoch curve during training in 5-class classification. We observe that the training curves in Figs. 6 and 7 converge properly with increasing epochs. Therefore, we conclude that our trained model is free from overfitting issues in both classifications.

We have also conducted an experiment to analyze whether the GRU layers give any extra performance benefits. Therefore, we have removed the GRU layers from our model pipeline and again evaluated our model using the same dataset. The comparisons of both results are tabulated in Table 4. We observe that all of the performance metrics have improved significantly while GRU layers are added. Without GRU layers, our model pipeline gives 98.00% accuracy in 2-class classification and 73.13% accuracy in 5-class classification. Therefore, we can see that there is a significant improvement in classification results when GRU layers are used.

**Fig. 8:**
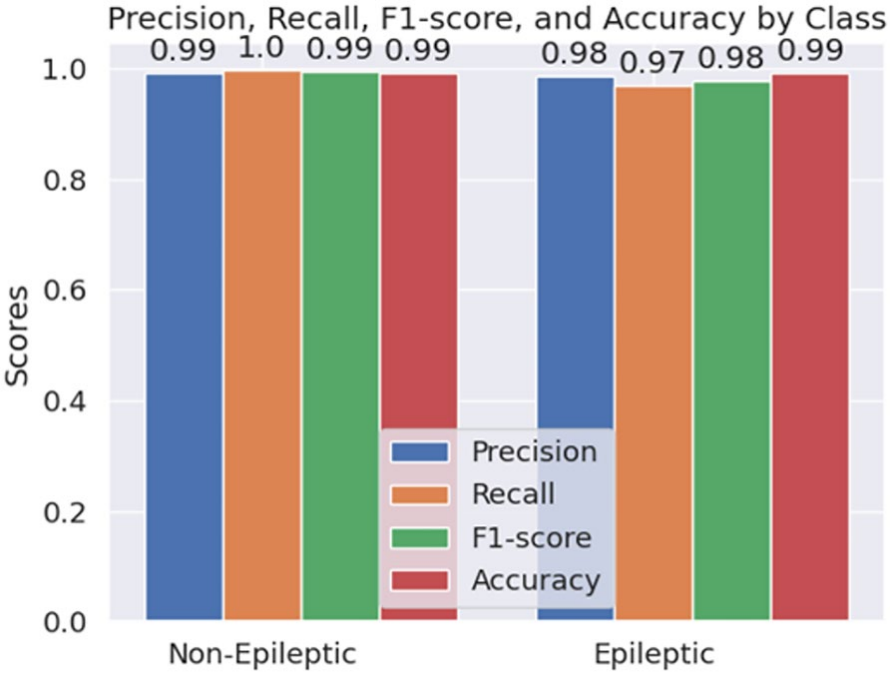
Bar chart showing the class-wise metric scores of our proposed HyEpiSeiD model for 2-class classification on UCI Epilepsy dataset

**Fig. 9:**
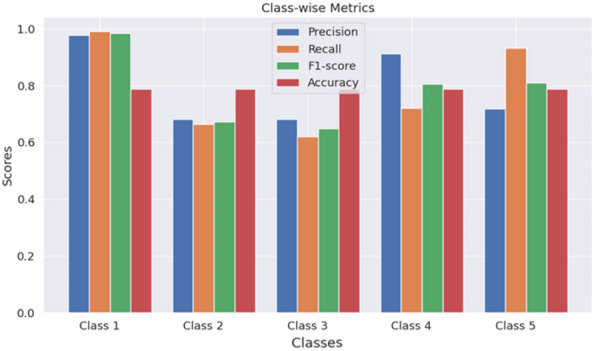
Bar chart showing the class-wise metric scores of our proposed HyEpiSeiD model for 5-class classification on UCI Epilepsy dataset

Figures 8 and 9 show class-wise evaluation metric scores considering 2-class and 5-class classification on the UCI Epilepsy dataset, respectively. We observe that for a 2-class classification, all the metric values, i.e. precision, recall, f1-score, and accuracy for each individual epileptic class, are around 98% to 99%. It means, our HyEpiSeiD model is not biased towards classifying a particular class. On the other hand, in case of 5-class classification, the metric values of class ’1’ are the highest. It means that our model can predict class ’1’ most accurately. But, the metric values for the remaining epileptic classes ’2’, ’3’, ’4’ and ’5’ are decent but less by a significant margin than class ’1’. Finally, we can conclude that our model framework performs very well on this dataset.

**Table 5:** 2-class classification results of our proposed HyEpiSeiD framework on Mendeley dataset

Dataset	Evaluation metric	Value
	Accuracy (%)	97.50
	Precision (%)	97.61
Mendeley dataset	Recall (%)	97.50
	F1-score (%)	97.49

**Fig. 10:**
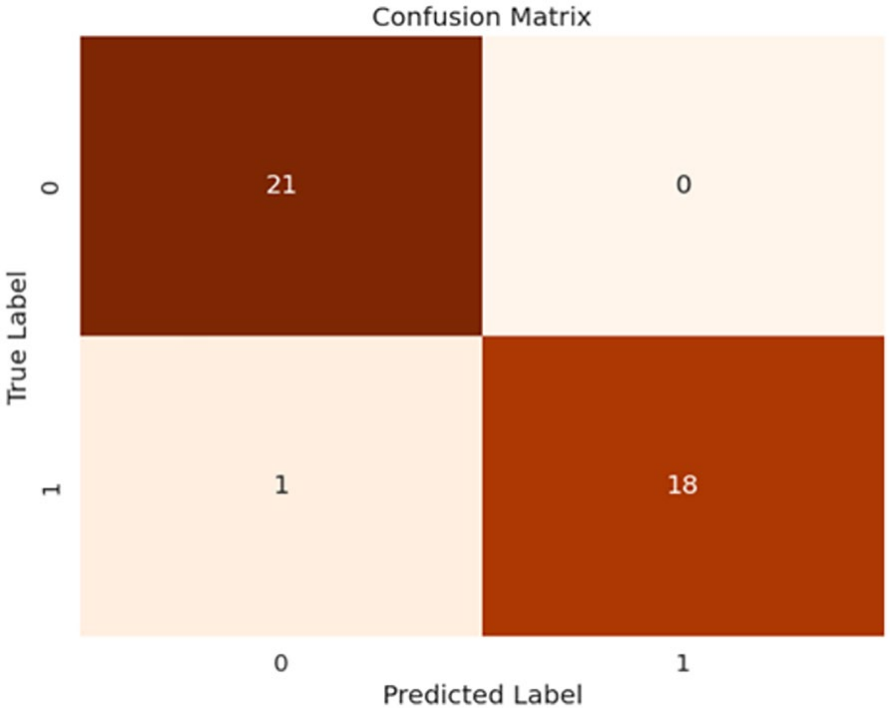
Confusion matrix of our proposed HyEpiSeiD model on Mendeley dataset for 2-class classification

**Fig. 11:**
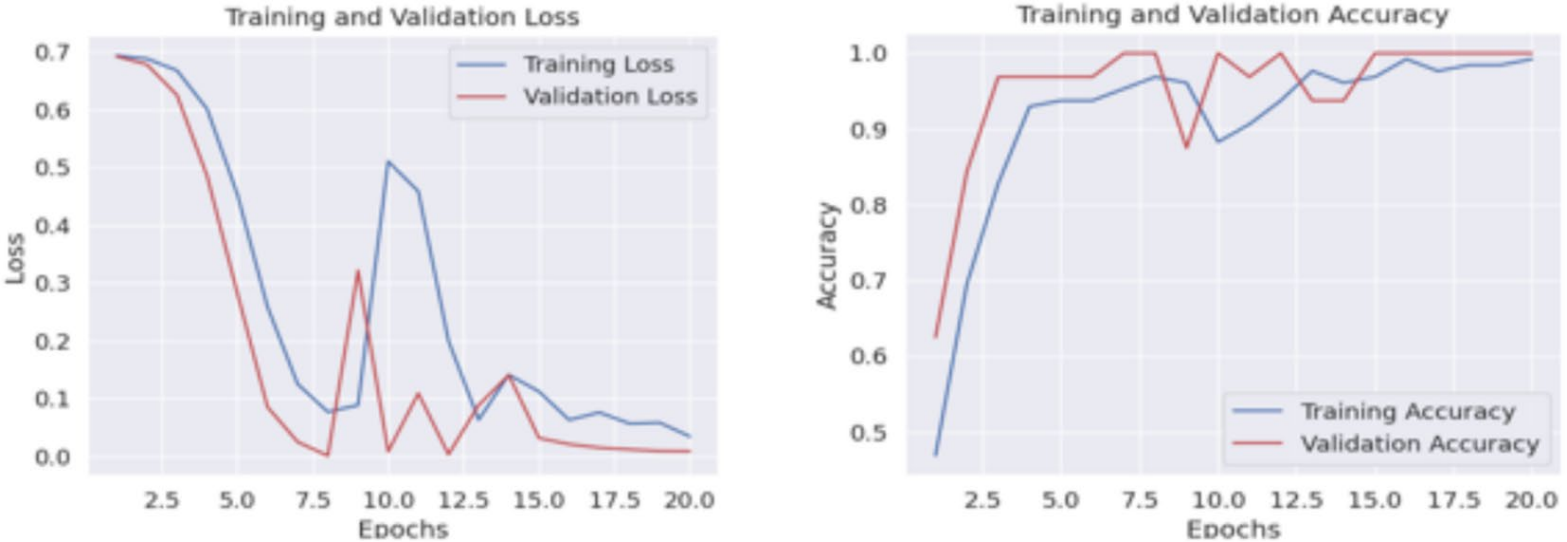
Loss vs epoch and accuracy vs epoch curve of our proposed HyEpiSeiD model on Mendeley dataset for 2-class classification

#### Results on mendeley dataset (Renuka Khati)

We have also evaluated our proposed HyEpiSeiD model pipeline on the Mendeley dataset (Renuka Khati). For this dataset, we split it into an 80: 20 train-test ratio and trained our model for 20 epochs. The batch size has been set to 32. In our case, 20% of the total training data is kept for validation. Unlike the UCI Epilepsy dataset, we have only performed 2-class classification here since the dataset consists of only 2 classes: healthy and seizures. Therefore, 5-class classification is not possible here.

We observe that our model achieves 99.22% training accuracy and 99.97% validation accuracy. Finally, our model gives 97.50% accuracy, 97.61% precision, 97.5% recall, and 97.49% f1-score on the test dataset. The evaluation metric results which we have obtained in this dataset are shown in Table 5. The confusion matrix gives the overall performance of our proposed framework on the dataset by showing the model’s true positive, true negative, false positive and false negative values. In our observation, the counts of positive and true negative entries are much higher than those of false positive and false negative entries. It indicates that our model performs very well regarding all possible evaluation metrics i.e. precision, recall, f1-score, accuracy. Figure 10 shows the confusion matrix we obtained for our proposed model evaluation on the Mendeley dataset. We also observe that both training curves, i.e. loss vs epoch and accuracy vs epoch in Fig. 11 converge properly at the end. This shows that the model is free from overfitting issues.

**Table 6:** 2-class classification comparison of our HyEpiSeiD model pipeline with GRU and without GRU on Mendeley dataset

Model	Accuracy	Precision	Recall	F1-score
With GRU	97.50%	97.61%	97.50%	97.49%
Without GRU	95.40%	96.61%	96.42%	96.51%

**Fig. 12:**
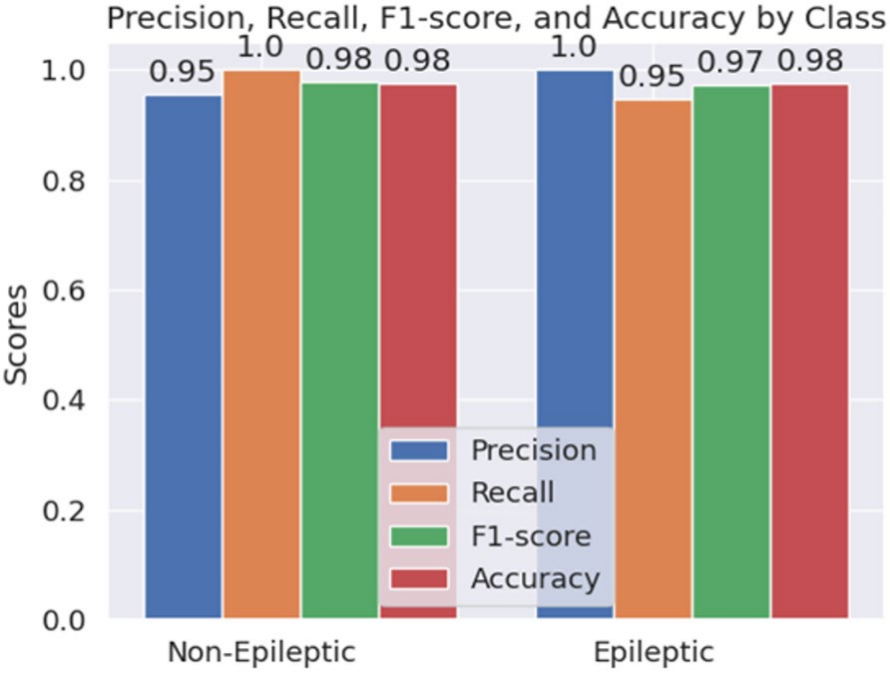
Bar chart of class-wise metric scores of our proposed HyEpiSeiD model for 2-class classification on Mendeley dataset

Like the UCI Epilepsy dataset, we have also conducted the same experiment to identify whether the GRU layers significantly enhance our model’s performance in the Mendeley dataset (Renuka Khati). We have first evaluated our model, including the GRU layers on the dataset. Then, we removed the GRU layers from our model pipeline and again evaluated them on the same dataset. Lastly, we have made a comparison between these two evaluation results. The comparison is shown in Table 6. We can see that GRU layers improve the model’s accuracy by 1.1%, precision by 1%, recall by 1.12% and f1-score by 0.98%. We can claim that GRU layers have improved our model’s performance for both datasets. Finally, we have performed a class-wise metric evaluation on the Mendeley dataset. A visual representation of these class-wise evaluation metric values for the Mendeley dataset is shown in Fig. 12. We can see that the values of each evaluation metric, i.e. precision, recall, accuracy, and f1-score for each individual class, are in the range from 95% to 100%. Each metric value is very high, indicating that the trained model has no strong bias towards classifying a particular class. Therefore, we can claim that our proposed HyEpiSeiD model is robust and will give high classification accuracy for any unknown dataset.

**Table 7:** *Comparison of our proposed HyEpiSeiD model framework against models pertaining to epilepsy recognition considering 2-class classification over the UCI dataset.*

References	Method	Accuracy (%)	Precision (%)	Recall (%)	F1-score (%)
Xu et al. [109]	CNN+LSTM	99.39	98.39	98.79	98.59
Rohan et al. [97]	ANN+XGBoost	98.26	–	–	–
Shankar et al. [98]	ANN+PCA	97.55	94.24	91.48	–
Rahman et al. [99]	SVM+MLP	97.27	96.93	94.53	95.63
Prakash et al. [100]	GRU	98.84	96.90	97.10	97.00
Raibag et al. [101]	SVM+RBF	96.00	–	–	–
Osman et al. [102]	SOM+RBF	97.47	–	–	–
Upadhyaya et al. [103]	RF+BAT	96.78	–	96.89	–
Woodbright et al. [104]	CNN	98.65	–	–	–
Wang et al. [105]	RF	98.96	–	–	–
**Bhadra et al.**	**HyEpiSeiD**	**99.01**	**99.01**	**99.04**	**99.02**

Bold values indicate the performance of our model

**Table 8:** Comparison of our proposed HyEpiSeiD model framework against models with and without pre-trained transfer learning CNN models pertaining to epilepsy recognition considering 5-class classification over the UCI dataset

References	Method	Accuracy (%)	Precision (%)	Recall (%)	F1-score (%)
Guha et al. [106]	ANN	78.00	77.00	76.00	72.00
Polat et al. [110]	SVM+MAD	76.70	–	–	–
Rohan et al. [97]	ANN+XGBoost	76.20	–	–	–
GoogleNet [111]	GoogleNet	77.97	–	–	–
AlexNet [112]	AlexNet	71.25	–	–	–
VGG16 [113]	VGG16	74.32	–	–	–
Mao et al. [114]	CNN+CWT	72.49	–	–	–
**Bhadra et al.**	**HyEpiSeiD**	**78.34**	**79.38**	**78.73**	**78.52**

Bold values indicate the performance of our model

### Comparison with existing methods

After evaluating our HyEpiSeiD model framework against two publicly available datasets, i.e., the UCI Epilepsy dataset and the Mendeley dataset (Renuka Khati), we have compared our model framework with some state-of-the-art methods which are used in epilepsy recognition tasks. Here, we have compared our proposed model with popular existing methods in Tables 7 and 8.

In Table 7, we have included only those articles where a 2-class classification has been performed on the UCI Epilepsy dataset. We have only compared the 2-class classification results of our model with theirs. Similarly, in Table 8, we have included only those articles where 5-class classification has been performed on the UCI Epilepsy dataset. We have only compared the 5-class classification results of our model with theirs. The comparison values for GoogleNet, AlexNet, and VGG16 are taken from [115].

Unfortunately, there is no such kind of epilepsy detection work currently available on the Mendeley dataset(Renuka Khati). Therefore, we could not put any comparison table for the Mendeley dataset like the UCI Epilepsy dataset. As a recent work on the Mendeley dataset, we can say that our model has performed pretty well, giving a decent 97.50% classification accuracy on this dataset.

## Conclusion

In this study, we have proposed a 1D CNN-GRU hybrid deep learning methodology, named as HyEpiSeiD, for automatically recognising epilepsy seizures from EEG data. We have evaluated our proposed HyEpiSeiD framework on (i) the UCI Epilepsy dataset and (ii) the Mendeley dataset by Renuka Khati. Our proposed hybrid model gives an overall 99.01% accuracy, 99.01% precision, 99.04% recall and 99.02% f1-score for the 2-class classification of the UCI dataset. Similarly, it also gives 78.34% accuracy, 79.38% precision, 78.73% recall, and 78.52% f1-score while considering the 5-class classification of the UCI dataset. Therefore, we can conclude that our proposed hybrid model framework has given robust classification results on the UCI dataset. Upon analysing the individual class-wise evaluation metric scores, it can be said that our model is not biased towards classifying a particular class. Like the UCI dataset, our proposed hybrid model has also been evaluated on the Mendeley dataset as well. It gives 97.50% classification accuracy on the Mendeley dataset. We can see that all individual class-wise evaluation metric scores for the Mendeley dataset are also very high. Therefore, our model is also not biased towards classifying a particular class. Upon observing the evaluation results obtained from these two datasets, we can conclude that our model framework has a robust classification ability for any general epilepsy dataset. Moreover, it has no strong bias towards classifying any particular class, making it a perfect model framework for epilepsy classification. For the betterment of the research community, the source code of our proposed model is made public, which can be downloaded from https://github.com/rajcodex/Epilepsy-Detection. In this study, we focus more on selecting a combination of models rather than the pre-processing procedure of raw EEG signal data.The datasets that have been used in this study, are already pre-processed.Therefore, in future, we aim work on a robust pre-processing framework for raw EEG signals. A robust and optimal pre-processing methodology can help us to extract proper features which can be further used in any model for epilepsy classification task.We can further explore recent state-of-the-arts computer vision models in epilepsy deection tasks.In that case, first, we have to pre-process EEG time-series data into images followed by employing a computer vision model.

## Data Availability

The data that support the findings of this study are openly available at: 1. https://archive.ics.uci.edu/dataset/388/epileptic+seizure+recognition 2. https://data.mendeley.com/datasets/k2mzn5zvyg/1.
